# Enabling routine β-thalassemia Prevention and Patient Management by scalable, combined Thalassemia and Hemochromatosis Mutation Analysis

**DOI:** 10.1186/s12881-020-01017-x

**Published:** 2020-05-15

**Authors:** Ghazala Hashmi, Asim Qidwai, Kristopher Fernandez, Michael Seul

**Affiliations:** 1 BioMolecular Analytics, 10 Independence Blvd, Suite 140, Warren, NJ 07059 USA; 2 Sanya K Hashmi Foundation, 56 Telegraph Hill Road, Holmdel, NJ 07733 USA; 3 Afzaal Memorial Thalassemia Foundation, Karachi, Pakistan

**Keywords:** Beta (β)-thalassemia, Hemochromatosis, Iron overload, Inherited disorders, LeanSequencing, Patient management, Prevention

## Abstract

**Background:**

Beta (β)-thalassemia is one of the most common inherited disorders worldwide, with high prevalence in the Mediterranean, the Middle East and South Asia. Over the past 40 years, awareness and prevention campaigns in many countries have greatly reduced the incidence of affected child births. In contrast, much remains to be done in South-Asia. Thus, for Pakistan, an estimated ~ 7000 children annually are born with thalassemia, with no sign of improvement. Although there is good agreement that intermarriage of carriers significantly contributes to the high prevalence of the disorder, effective tools for molecular screening and diagnosis on which to base prevention programs are not readily available.

**Methods:**

Here, we present results for a novel LeanSequencing™ process to identify a combination of 18 β-thalassemia mutations (including the sickle cell anemia mutation, HbS, and structural variants HbC and HbE) and 2 hemochromatosis mutations in a multi-ethnic population of 274 pediatric and adolescent patients treated at Afzaal Memorial Thalassemia Foundation in Karachi, Pakistan.

**Results:**

We found substantial differences in the predominance of disease-causing mutations among the principal ethnic groups in our cohort. We also found the hemochromatosis mutation H63D C > G in 61 (or 22.1%) of our patients including 6 (or 2.2%) homozygotes.

**Conclusions:**

To our knowledge, this is the first screen combining a large set of β-thalassemia and hemochromatosis mutations, so as to facilitate the early identification of patients who may be at increased potential risk for complications from iron overload and thereby to improve the prospective management of thalassemia patients.

## Background

Beta (β)-thalassemia is a genetic disorder that remains a public health challenge in many countries around the world, with a high prevalence especially in the Mediterranean, Middle East and South as well as South-East Asia. Its molecular origin lies in abnormalities of the beta (β)-globin gene which leads to the limited synthesis or complete lack of correctly folded hemoglobin (“HBB”) [1–3]. Individuals who inherit 2 abnormal copies of the β-globin gene develop anemia and often require life-long red cell transfusion support to maintain adequate hemoglobin levels. The nature and severity of clinical symptoms are known to reflect the specific underlying mutation(s), and even individuals with just a single abnormal copy of the gene may be affected, though generally with less severe clinical manifestations [3, 4]. Given the autosomal recessive pattern of inheritance, pre-marital carrier screening can be effective in prevention [5], and mandatory programs have been introduced in several of the affected countries [6]. In addition, the early identification of the specific underlying pathogenic mutations by pre-natal and neo-natal screening, likewise implemented in many countries [7], represents a critical element of patient management.

Thalassemia remains prevalent in Pakistan where, according to current estimates, 5000–7000 children are born each year with thalassemia major [8]. Several organizations provide information to raise awareness and offer programs to diagnose the disorder [8–10]. However, while at least two provincial governments, Sindh and Punjab, to date have passed bills to support awareness campaigns and premarital carrier screening, a national program remains to be implemented. To a large extent, diagnosis currently relies on electrophoresis which, while relatively inexpensive, does not provide the differential diagnosis available only by the reliable identification of the underlying mutations. In fact, many patients are misdiagnosed for anemia. At present, the requisite genetic testing is performed only sporadically, as it is perceived to be costly, and in any case, is currently available only at a few hospital laboratories [8, 10]. Thus, there is growing agreement that limited access to genetic testing and reliable disease diagnosis in fact is contributing to a gradual increase in the number of thalassemia patients in the country.

When diagnosed, clinical treatment, in addition to addressing acute clinical complications, includes the transfusion of red cells [11, 12]. However, transfusion, often on chronic protocols, frequently leads to the formation of allo-antibodies as well as the accumulation of excess iron [13] which accumulates in tissues and organs and may lead to major organ damage. Patients with HFE (“high-Fe”) gene mutations that cause hemochromatosis may be at increased risk [14], but, at present, even the scarce genetic analysis performed on thalassemia patients in Pakistan and elsewhere generally is limited to mutations in the HBB gene, and does not routinely include mutations in the HFE gene.

Here, we describe a novel approach to simultaneously detect a set of 18 HBB mutations, as well as 2 HFE mutations in chronically transfused pediatric and adolescent thalassemia patients, including the determination of the abundance of these mutations in several ethnic groups in Pakistan. Our ultimate objective is that of providing a cost-effective screening process that holds the promise of improving thalassemia prevention and patient management.

## Methods

### Patient cohort

Our analysis included samples from a total of 288 (mostly) pediatric and adolescent patients of diverse (self-identified) ethnic background, most abundant among them Urdu-speaking (North India), Sindhi, Saraiki, Punjabi, Pathan and Balochi, all treated at the Afzaal Memorial Thalassemia Foundation (“AMTF”) hospital in Karachi. All patients were on chronic transfusion protocols.

The majority of these patients (*n* = 275) had a diagnosis of β-thalassemia, established by standard clinical methods including blood work, hemoglobin electrophoresis and HPLC; in addition, 13 blinded samples from patients with diagnoses other than β-thalassemia also were included as “negative controls”, namely: hereditary spherocytosis (5), immune thrombocytopenic purpura (“ITP”) (2), hemolytic anemia (2), autoimmune hereditary anemia (1), Fanconi anemia (1), bone marrow failure syndrome (1) and alpha (α)-thalassemia (1). One sample failed to produce data, both by LeanSequencing and Sanger sequencing, and was excluded from further analysis. Of the remaining 274 patients with β-thalassemia, 132 were female, with a mean age of 8.7 years, and 142 were male, with a mean age of 8.6 years (Table 1); the age distribution for both is positively skewed (with a mode below the mean, near ~ 6 years).

**Table 1:** Patient Demographics

Ethnicities in Patient Cohort			Age Distribution		
	Count	Percentage	Yrs	F	M
Pathan	74	27.0%	1	3	3
Urdu	45	16.4%	2	7	5
Saraiki	41	15.0%	4	19	26
Sindhi	31	11.3%	6	33	29
Balochi	23	8.4%	8	17	14
Punjabi	15	5.5%	10	20	27
Memon	11	4.0%	12	14	16
Hazarwi	9	3.3%	14	6	6
NA	9	3.3%	16	5	9
Burohi	5	1.8%	18	1	4
Bhatti	3	1.1%	20	2	2
Afreedi	2	0.7%	22	2	1
Bangali	2	0.7%	24	1	0
Hindko	2	0.7%	26	0	0
Mansahra	1	0.4%	28	1	0
Mianwali	1	0.4%	30	0	0
			32	1	0

Self-declared ethnicities (left) and age distribution by gender (right); NA refers to individuals whose ethnicity was not recorded

### Sample collection & processing

Duplicate barcoded buccal swab samples were collected, and crude extracts prepared using the Phusion Human Specimen Direct PCR kit (ThermoFisher Scientific, Waltham, MA) by a protocol that, following preparation of the lysate, requires only simple spinning (but no high-speed centrifugation) and takes ~ 10 min to complete for a batch of 8 samples. These extracts were processed by a multiplexed PCR reaction comprising both HBB and HFE genes, followed by an allele-specific labeling reaction and analysis by capillary electrophoresis (the latter performed by send-out to a service provider, Genewiz, South Plainfield, NJ), in accordance with LeanSequencing™ (“LSQ”).

### LSQ protocol: amplify & discriminate

**Fig. 1:**
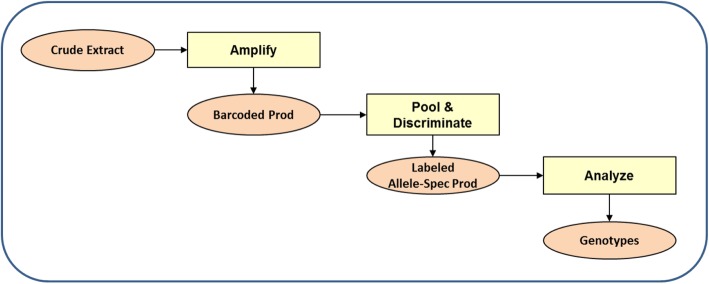
Lean Sequencing Process Flow

LeanSequencing is a novel process developed at BioMolecular Analytics (Warren, NJ) for analyzing sequence variants. The two analytical steps in LeanSequencing are amplification and discrimination (Fig. 1). Amplification, in a single multiplex PCR reaction, produces a set of amplicons comprising all β-thalassemia and hemochromatosis sequence variants of interest; each amplicon bears a molecular tag (aka “barcode”) that identifies the sample of origin and permits amplicons from multiple samples to be combined (“pooled”). Following pooling, discrimination, in a second multiplex PCR reaction, produces labeled allele-specific amplicons, from, in this case, 4 samples per well, for analysis in a standard capillary sequencer, in this case the 96-channel ABI 3730xl.

The protocol omits extraneous steps including DNA purification, “normalization” and “clean-up” reactions. The discrimination reaction is configurable so as to select specific marker (“SNP”) sets according to ethnicity or geographic area of interest, and to accommodate individual or multiple (2 or 4) “pooled” samples per well. The entire process takes less than 1 h of hands-on time per 96-well plate, and it may be readily automated using inexpensive laboratory pipetting instrumentation. The process achieves a very high data rate – for example, in the “pools-of-4” configuration used here, a single run on a standard sequencer with a 96-capillary array produces complete molecular HBB and HFE profiles for 384 samples. Raw data, in the form of sequence traces, can be uploaded to the BioMolecular Analytics web portal for processing by proprietary software that can be accessed from anywhere using only a web browser.

### Selection of HBB and HFE variants

To select mutations of interest, we started with the set most commonly observed in Pakistan and Middle Eastern countries [15], namely: –del 619; IVS I-1 G > T or G > A; IVS I-5 G > C; IVS I-6 T > C, IVS I-110 G > A, − 88 C > T, − 29 A > G, cd 8/9 + G, cd 41/42 –TTCT, as well as cd 6 A > T (the sickle cell anemia mutation, “HbS”) and the two structural variants, cd 6 A > G (“HbC”) and cd 26 G > A (“HbE”). Following initial testing of this design, we selected 64 samples with at most one of these most commonly observed mutations for Sanger sequencing of HBB gene exon 1, partial intron 1 and exon 2 to check for any additional mutations or variants, notably these five mutations: cd 5 –CT, cd 15 G > A, cd 16 –C, cd 30 G > C (“Monroe”), and the rare − 90 C > T mutation (aka rs34999973 C > T [16]), as well as these two variants: rs713040 c.9 T > C and rs35799536 G > C .

The final selection for our LSQ application comprises 18 HBB and 2 HFE mutations excluding the two variants (Table 2); as initial testing showed all patients to be normal for S65C, this was omitted. This selection covers many of the mutations commonly observed in other regions, namely (with reference to Tb. 1 in reference [2] and Fig. 3 in reference [3]): Mediterranean (cd 5 –CT, IVS I–1 G > A, IVS I–6 T > C, IVS I–110 G > A); Central and SE Asian (cd 41/42 –TTCT); East Asian (IVS I–5 G > C); African (− 29 A > G, − 88 C > T); and Indian (−del 619) and (with reference to Tb. 1 in [18]): Middle Eastern (IVS I–5 G > C, IVS I-1 G > A, IVS I-6 T > C and cd 5 –CT).

**Table 2:**
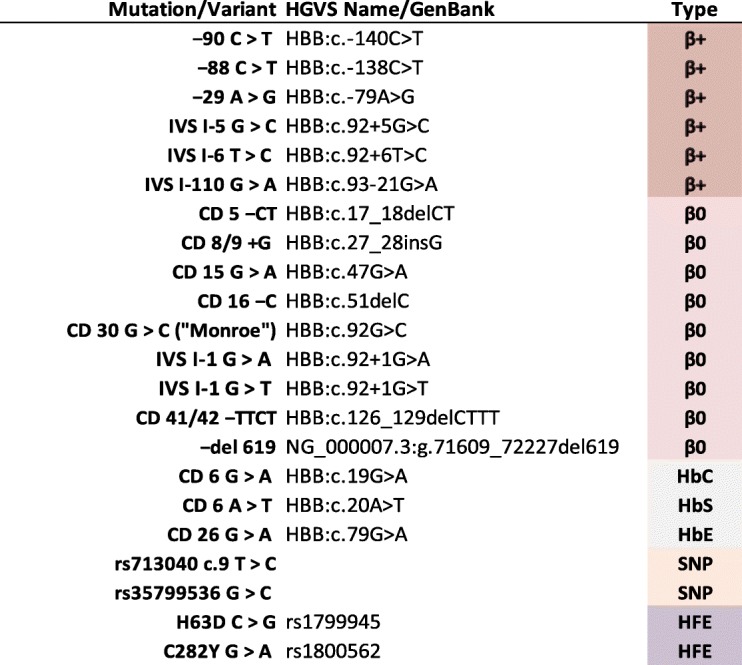
Mutations and Variants Analyzed

Mutation types β(+) and β(0) are in accordance with http://globin.cse.psu.edu/ [17]; the HBB variants marked “SNP” were not included in the final LSQ selection

### Statistical analysis

Allele frequencies were determined from genotypes by “gene counting”. All analysis was performed, and all data tables and figures were generated, using Microsoft Excel (2010).

## Results

### Overall abundance of mutations

Table 3 lists estimated variant allele frequencies for the subset of HBB and HFE mutations encountered in our cohort. The frequencies of the six most abundant β-thalassemia mutations overall – which are further analyzed by principal ethnic group - exceeded 10% in frequency, and among these, the three most abundant, exceeded 20%, namely: IVS I-5 G > C (42.0%); cd 8/9 + G (27.9%) cd 30 G > C (“Monroe”, 22.3%).

**Table 3:** Pan-ethnic Frequencies of Observed HBB and HFE Mutations & Variants

Name	IVS I-5 G > C	CD 8/9 + G	CD 30 G > C (“Monroe”)	CD 15 G > A	CD 16 -C	CD 5 -CT	CD 41/42 -TTCT	IVS I-1 G > T or G > A	del-619	− 90 C > T	CD 26 G > A	CD 6 A > T	rs713040 c.9 T > C	rs35799536 G > C	H63D C > G
Type	β(+)	β(0)	β(0)	β(0)	β(0)	β(0)	β(0)	β(0)	β(0)	β(+)	HbE	HbS	SNP	SNP	HFE
Freq	0.420	0.279	0.223	0.163	0.141	0.109	0.068	0.033	0.033	0.022	0.007	0.002	0.256	0.222	0.123

Less commonly, we also observed structural mutations, namely: cd 26 G > A (HbE) (0.7%, corresponding to 4 heterozygotes); and cd 6 A > T (HbS) (1 heterozygote); we determined one patient to be homozygous for the rare − 90 (aka rs34999973) C > T mutation, without any other mutation or variant, and we found one additional patient who was compound heterozygous for rs35133315 del T (an additional variant detected by Sanger sequencing, but not included in the LSQ design) and IVS I-5 G > C. None of the individuals in our cohort was a carrier for any of four additional mutations (− 88 C > T and − 29 A > G, IVS I-6 T > C, IVS I-110 G > A or the HbC variant), though these have been observed in other ethnic groups outside of Pakistan.

### Variants of unknown significance

The two variants, rs713040 c.9 T > C and rs35799536 G > T, were quite prevalent in β-thalassemia patients as well as in individuals with diagnoses other than β-thalassemia. Thus, 10 of the former had one or both of these variants in combination with a pathogenic mutation. In contrast, 10 of the latter had one or more of the variants, but none of the mutations.

### Overall mutation status for patients and negative controls

The configuration of mutations and that of the phenotypes observed in our patient cohort are summarized in Table 4. As expected, the majority of the patients in our cohort were homozygous (*n* = 213 of 274, or 77.7%) or compound heterozygous (*n* = 37 or 13.5%) for β-thalassemia mutations. Among the homozygotes, roughly equal numbers were homozygous for β(+) (*n* = 97) and β(0) (*n* = 116) mutations, with IV I-5 G > C predominating among the former.

**Table 4:** Configuration of Observed β-Thalassemia Mutations

Zygosity	Phenotype	Count	Cum	Cum (%)
**Homozygous**	**β+ / β+**	**97**	**97**	**0.354**
	**β0 / β0**	**116**	**213**	**0.777**
**cHet**	**β+ / β0**	**26**	**239**	**0.872**
	**β0 / β0’**	**11**	**250**	**0.912**
**Het or cHet w/ SNP**	**β+ / HbS, C, E**	**4**	**254**	**0.927**
	**β+ / (SNP)**	**6**	**260**	**0.949**
	**β0 / (SNP)**	**13**	**273**	**0.996**
**Other**	**HbE / SNP**	**1**	**274**	**1.000**

“Het” and “cHet” denote “heterozygous” and “compound heterozygous”, respectively, where β(0)/β(0)’ refers to a compound heterozygous configuration comprising 2 different β(0) mutations; “(SNP)” indicates a mutation in possible combination with one of the variants described in the text

An additional 23 individuals (or 8.4%) had 1 of the mutations, namely 10 in isolation, 9 in combination with one of the non-pathogenic variants, and 4 in combination with one of the structural variants (including one who was compound heterozygous for IVS I-5 G > C and HbS). One patient was homozygous for both SNPs and had the HbE structural variant; but for this individual, all patients had at least 1 of the mutations included in our design. We confirmed that 10 of the 13 “negative controls” had at least one copy of one or both of the variants rs713040 c.9 T > C and rs35799536, but none of the pathogenic mutations, while 3 had neither.

### Predominance of different disease-causing mutations in principal ethnicities

**Fig. 2:**
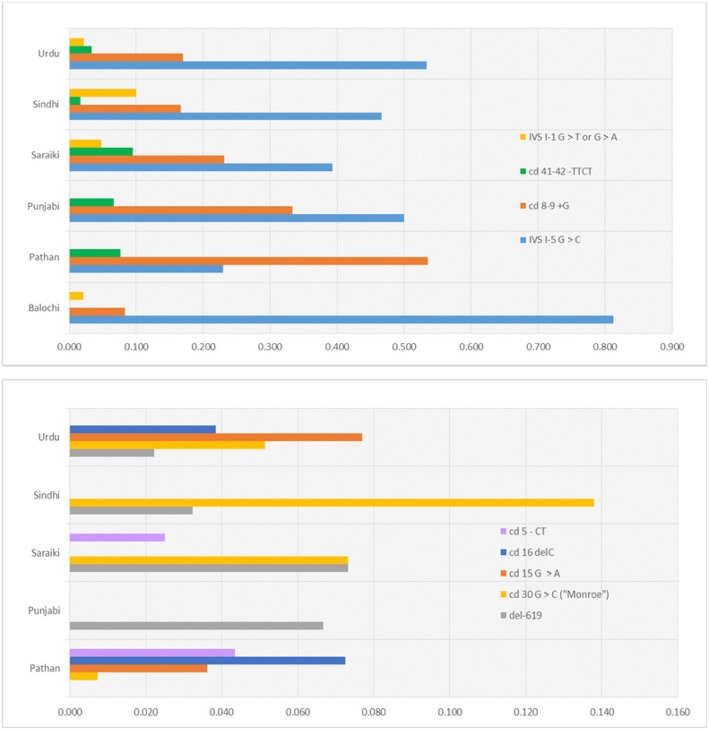
Mutation Frequencies in the Most Prevalent Ethnic Groups In Our Cohort. Top: Locally predominant mutations; Bottom: Less abundant mutations; none of these were observed in Balochi patients (not shown)

As evident from Fig. 2, and from a related Supplemental Table giving genotype counts, the prevalence of the disease-causing mutations varies substantially across the principal ethnic groups in our cohort, namely: Pathan (74), Urdu-speaking (45), Saraiki (41), Sindhi (31), Balochi (23) and Punjabi (15).

Thus, a remarkable 18 out of the 23 Balochi patients, or almost 80%, were homozygous, and one additional patient heterozygous for IVS I-5 G > C which, in combination with cd 8/9 + G, accounted for 90% of the mutations observed in this group; 33 of the 74 Pathan patients, or 42%, were homozygous for cd 8/9 + G, and an additional 12 were heterozygous for that mutation; a significantly higher proportion of the Sindhi patients compared to that in other groups, namely 2 of 31, were homozygous, and an additional 2 were heterozygous, for IVS I-1 G > T; and a higher proportion of the Saraiki patients, compared to that in other groups, namely 3 of 41, had the del619 deletion.

### Hemochromatosis

**Fig. 3:**
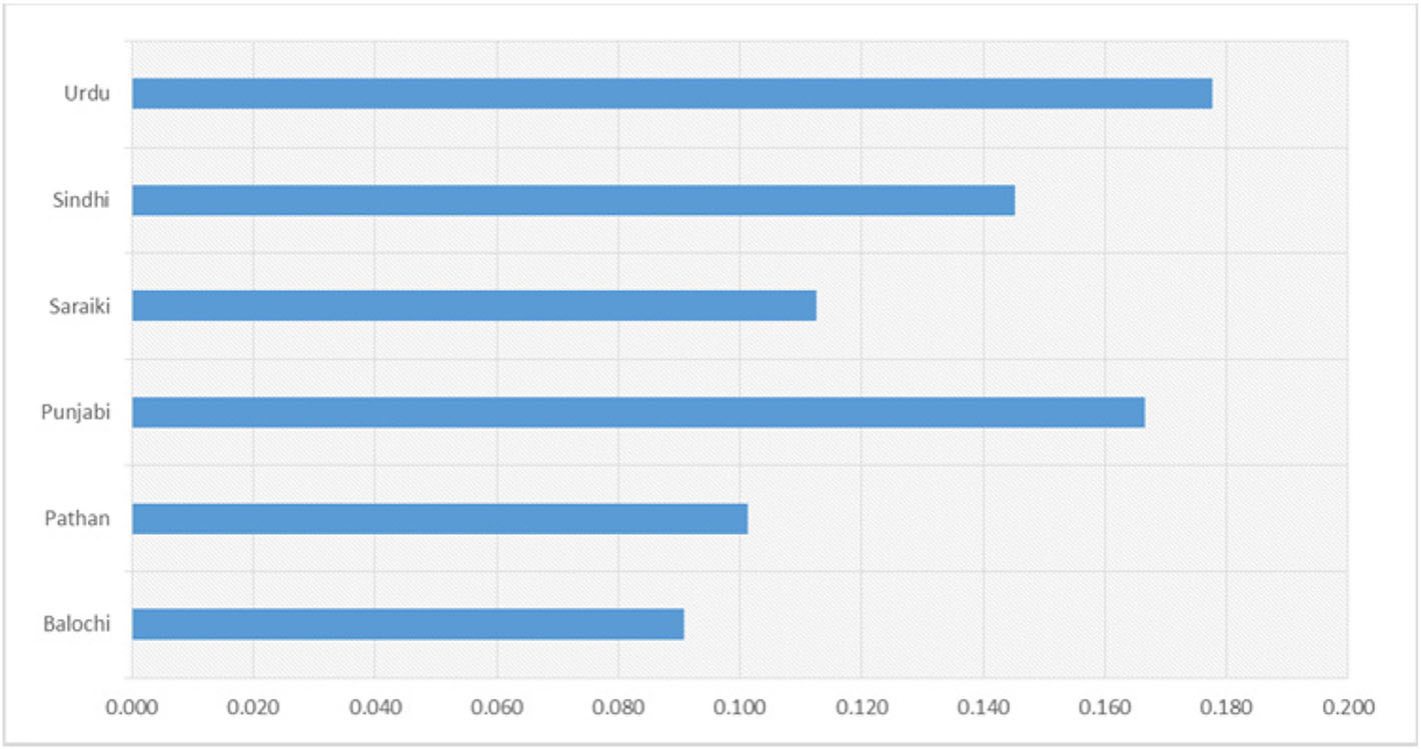
H63D variant allele frequency for principal ethnicities in our patient cohort

Of the patients in our cohort, 61 (or 22.1%) had at least 1 copy of the H63D C > G mutation which is strongly associated with this disorder: of these, 6 (or 2.2%) were homozygous; all patients were normal for C282Y (not shown). The frequency of the H63D variant allele exceeded 10% in all but one of the ethnic groups most abundantly represented in our sample, ranging from 9.6% (Pathan) to 17.8% (Urdu), as summarized in Fig. 3 and in a related Supplemental Table giving genotype counts. To our knowledge, the high prevalence of this mutation in a Pakistani population of β-thalassemia patients has not been previously reported.

## Discussion

Ours appears to be the first study of combined β-thalassemia and hemochromatosis mutation prevalence focusing exclusively on patients, and it thus complements a recent study by Yasmeen and colleagues [19] , namely by: enrolling additional ethnicities; extending the coverage of β-thalassemia, and concurrently probing hemochromatosis mutations; and accommodating a simple buccal swab sample collection with a protocol in the context of our novel LeanSequencing ("LSQ") process that is particularly well suited to large scale screening. A previous related study also included putative carriers, that is: individuals having an affected family member, along with chorionic villus samples from referrals by thalassemia clinics [20].

As expected, the majority of our patients, namely 250 (or 91.2%), were either homozygous or compound heterozygous for the mutations in our design. However, identity and abundance of the predominant mutation(s) differ markedly among the six principal ethnic groups represented in our cohort.

Our analysis indicates that our selection, comprising 18 HBB (“hemoglobin”) mutations, as well as 2 HFE (“hemochromatosis”) mutations, provides excellent coverage: all but one of the patients in our multi-ethnic cohort of 274 (predominantly) pediatric and adolescent Pakistani β-thalassemia patients in fact had at least 1 of the selected mutations (Table 1). This selection also achieved reliable differential analysis: of the 13 individuals with diagnoses other than β-thalassemia, none had any of the mutations, but only common variants, while 3 (namely 2 diagnosed with hereditary spherocytosis and one diagnosed with ITP) had neither variants nor mutations.

Regarding coverage in other affected countries and regions, the selection also contains mutations that, while rare or absent in our cohort, are observed in other populations. As LSQ is readily configurable, at the discrimination stage, it may be extended by “reading-out” additional mutations such as cd 39 C > T (abundant in Spain, Sardinia, Middle East and North-Africa).

We identified six H63D homozygotes in our patient cohort, corresponding to an estimated prevalence of 2.2%, with a variant allele frequency exceeding 10% in all but one the principal ethnic groups represented in our study, in contrast, for example, to a previous report concerning a North Indian population [21, 22].

In (otherwise) healthy individuals, H63D homozygotes have been reported to display increased transferrin saturation and serum ferritin, but decreased unsaturated iron binding capacity compared to H63D heterozygotes or normal individuals [23]. Our observation therefore naturally raises questions as to a potential adverse effect of hemochromatosis in β-thalassemia patients, namely to increase the risk of iron overload, and while hemochromatosis may confer elevated risk to all patients, the situation may be exacerbated by chronic transfusion. In fact, a recent report from Egypt does point to a correlation between homozygosity for that mutation and elevated levels of several clinical indicators of iron overload, including serum iron and ferritin [14]; others have found no correlation ([24], see also Kaur et al., ref. [21]).

A preliminary review of serum ferritin levels, for a subset of 124 of our patients, showed high levels in all by the time first readings were taken, suggesting that, regardless of a possible genetic predisposition to iron overload as a result of the H63D mutation, ferritin levels may reflect the cumulative adverse effect of a high number of transfusions, even during the first year of transfusion support. However, by including the most relevant HFE mutations in our β-thalassemia design, we will be able to spot patients with this potential additional risk factor, enabling a prospective study of new β-thalassemia patients with and without HFE mutations as the basis for a systematic evaluation of any statistically significant differences in ferritin levels and related parameters between patients with β-thalassemia and those with other diagnoses, and, among the former, those receiving and those not receiving periodic transfusions. HFE mutation status may provide an important factor in early risk assessment, to be taken into account when tailoring transfusion protocols and chelation therapy to individual patients so as to minimize the risk of iron overload.

## Conclusion

Programs for pre-marital, prenatal and neonatal screening for β-thalassemia which have been shown to yield substantial benefits [7] also hold promise for Pakistan, perhaps with a focus on the extended families of patients [9]. A recently published study reports a tendency for couples to abort when disease-causing mutations are detected in the foetus [8]. In addition, neonatal screening would provide the basis for interceding as early as possible, ideally by devising personalized transfusion protocols so as to minimize the systemic adverse consequences of iron accumulation.

In view of the results reported here, we believe that LeanSequencing offers an effective new approach to faithfully providing combined β-thalassemia and hemochromatosis screening and disease diagnosis to the larger population. The SKH and AMT foundations are planning to initiate this service in Pakistan and elsewhere.

## Supplementary information

**Additional file 1.**

## Data Availability

All genotype data extracted from sequencing traces and analyzed during this study are incorporated in this published article [and a supplementary table]. Data generated and analyzed for the current study are available from the corresponding author upon reasonable request.

## Abbreviations

LSQ: LeanSequencingTM
HFE: Hemochromatosis
HBB: Hemoglobin
AMTF: Afzaal Memorial Thalassemia Foundation
SKH: Sanya Kiran Hashmi Foundation
